# Differential Impact of Education on Gray Matter Volume According to Sex in Cognitively Normal Older Adults: Whole Brain Surface-Based Morphometry

**DOI:** 10.3389/fpsyt.2021.644148

**Published:** 2021-03-05

**Authors:** Dong Woo Kang, Sheng-Min Wang, Hae-Ran Na, Nak-Young Kim, Hyun Kook Lim, Chang Uk Lee

**Affiliations:** ^1^Department of Psychiatry, Seoul St. Mary's Hospital, College of Medicine, The Catholic University of Korea, Seoul, South Korea; ^2^Department of Psychiatry, Yeouido St. Mary's Hospital, College of Medicine, The Catholic University of Korea, Seoul, South Korea

**Keywords:** education, sex, gray matter volume, cognitively normal older adults, surface-based morphometry

## Abstract

**Background:** The effect of educational status on brain structural measurements depends on demographic and clinical factors in cognitively healthy older adults.

**Objectives:** The current study aimed to evaluate the impact of interaction between years of education and sex on gray matter volume and to investigate whether cortical volume has a differential impact on cognitive function according to sex.

**Methods:** One hundred twenty-one subjects between 60 and 85 years old were included in this study. Gray matter volume was evaluated by whole brain surface-based morphometry. Multiple regression analysis was used to analyze the effects of sex-cortical volume interactions on cognitive functions.

**Results:** There was a significant interaction between years of education and sex on the cortical volume of the left inferior temporal gyrus after adjusting for age, APOE ε4 allele prevalence, and total intracranial volume. In addition, we found a significant impact of the interaction between adjusted left inferior temporal volume and sex on CERAD-K total scores.

**Conclusion:** These findings have significant implications for the understanding of how sex could affect the role of cognitive reserve for cortical atrophy in cognitively intact older adults.

## Introduction

The concept of reserve has emerged to explain the differential impact of neuropathological changes due to the aging process and neurodegenerative disease on individual clinical outcome, including cognitive decline and decreased ability to perform daily functions (1). Brain reserves, which are based on the brain's quantitative characteristics, and cognitive reserves, which focus on the brain's adaptive functions, are the two broad classifications of the reserve (2). The cognitive reserve has been reported to play a neuroprotective and compensatory role for neuropathologic change accompanying the aging process (3). Educational status, one of the representative proxies of cognitive reserve, can be evaluated as an objective number that represents the ease of acquiring information (4, 5). Previous studies have demonstrated that fewer years of education increase the prevalence of dementia (6), and higher levels of education delay the occurrence of Alzheimer's disease (AD) through tolerance against the neuropathologic change of AD (7).

Among the brain's structural parameters, gray matter volume is a representative surrogate reflecting the aging process and pathological change (8, 9). Years of education have also been correlated with the gray matter volume of several brain regions, including the medial and frontal gyri, superior temporal gyrus, inferior parietal lobule, and the anterior and posterior cingulate cortex in older adults with normal cognitive function (10–12). Moreover, another previous study has reported the interaction between education and total brain volume on cognitive decline in cognitively intact older adults (13).

Prior studies have reported that the effect of educational status on brain structural measurements depends on demographic and clinical factors in older adults with healthy cognitive function. Age has been shown to interact with education in relation to cortical gray matter volume in the left middle frontal gyrus and right medial cingulate cortex (12). In addition, the impact of education on hippocampal volume differs according to the presence of amyloid-beta (Aβ) retention, and the degree of Aβ deposition has been associated with cortical volume (14).

Available evidence has described the effect of sex on cortical volume in cognitively normal older adults. Female subjects are shown to have greater superior temporal, inferior frontal gyrus, and cingulate cortex volume; male subjects demonstrate increased cortical volume in bilateral temporal lobes and entorhinal and perirhinal cortices (15). Additionally, the relevance of sex for age-related changes in cortical volume has also been documented (16). Whereas, males showed greater volume loss in frontal and temporal lobes, females displayed greater atrophy of the hippocampus and parietal lobe. Despite the significant effects of sex on age-related volume loss, few studies have determined whether the effect of education on gray matter volume differs according to sex.

The current study aimed to evaluate the impact of the interaction between years of education and sex on gray matter volume by whole brain analysis in cognitively intact older adults. Additionally, we set out to investigate whether the cortical volume of brain regions showing significant interaction had a differential impact on cognitive function according to sex.

## Materials and Methods

### Participants

One hundred twenty-one subjects between 60 and 85 years old were included in this study. Subjects were recruited from the Catholic Brain Health Center MRI database, which was built through the outpatient psycho-geriatric clinic of Yeouido Saint Mary's Hospital located in Seoul, Republic of Korea, from October 2018 through December 2020. The cognitive function of all subjects was assessed using the Korean version of the Consortium to Establish a Registry for Alzheimer's Disease (CERAD-K) (17). Measures included assessment in the Korean version of the Mini-Mental State Examination (MMSE-K) (18), Word List Memory (WLM), Word List Recall (WLR), Word List Recognition (WLRc). In addition, total memory (TM) domain scores were obtained by summing scores from the CERAD-K, WLM, WLR, and WLRc. Total CERAD-K scores were calculated by summing all CERAD-K subcategory scores, excluding the MMSE-K score. Details surrounding the usage of specific tests and the reviewing process are described in Supplementary Material. Inclusion criteria were as follows: (1) Participants with or without subjective memory complaints, beyond what would be expected for age, (2) Normal memory function, quantified by scoring above age, sex, and education adjusted cutoffs on the WLM, WLR, and WLRc domain, (3) MMSE-K score between 24 and 30, (4) Clinical Dementia Rating score of 0, (5) Memory Box score of 0, and (6) normal cognitive function, based on the absence of significant impairment in cognitive functions or activities of daily living. We excluded participants with any history of alcoholism, drug abuse, head trauma, or psychiatric disorders, those taking any psychotropic medications (e.g., cholinesterase inhibitors, antidepressants, benzodiazepines, and antipsychotics), those with multiple vascular risk factors, and those with extensive cerebrovascular disease. T2-weighted fluid-attenuated inversion recovery (FLAIR) data were acquired to objectively exclude vascular lesions or other diseases. The study was conducted under the ethical and safety guidelines set forth by the Institutional Review Board of The Catholic University of Korea, which approved all research activity. Informed and written consent was obtained from all participants.

### Data Acquisition and Preparation

Imaging data from the Catholic Brain Health Center MRI database were collected by the Department of Radiology of Yeouido Saint Mary's Hospital at the Catholic University of Korea using a 3 T Siemens Skyra MRI machine and a 32-channel Siemens head coil (Siemens Medical Solutions, Erlangen, Germany). One hundred twenty-one subjects were imaged with the T1-weighted magnetization-prepared rapid gradient-echo (MP-RAGE) sequence using the following parameters: image size = 224 × 224 × 256, voxel size = 0.9 × 0.9 × 0.9 mm^3^, repetition time (TR) = 1,940 ms, echo time (TE) = 2.6 ms, and flip angle = 9°. In addition, FLAIR data were collected using a 2D fast spin-echo sequence with the following parameters: TE = 76 ms, TR = 9 s, inversion time = 2.5 s, FOV = 22 cm × 22 cm, 42 oblique axial slices, slice thickness = 5 mm, image size = 224 × 224 × 256.

### Image Processing

FreeSurfer software (version 7.1.0, https://surfer.nmr.mgh.harvard.edu) was used to reconstruct and co-register the cortical surfaces and estimate brain structural features including cortical thickness, cortical volume, and surface area. Image processing for the cortical model was done in the following order: removal of non-brain tissue using a hybrid watershed algorithm (19), bias field correction, automated Talairach transformation, segmentation of subcortical white matter and deep gray matter structures (20, 21), intensity normalization, tessellation of the gray/white matter boundary and gray/cerebrospinal fluid (CSF) boundary, automated topology correction (19, 22), and surface deformation following intensity gradients to optimally place the gray/white and gray/CSF borders at the location where the greatest shift in intensity defines the transition to the other tissue class (23). Individual cortical folding patterns were then registered to a spherical atlas in order to match cortical geometry across subjects. Thickness was calculated at each location of the cortex as the distance between white matter and the pial surface (23). Procedures for the measurement of cortical thickness have been validated against histological analysis and manual measurements (24, 25). The cortical volume is defined as the product of cortical thickness and area. All data were smoothed with a 10 mm full width half maximum (FWHM) Gaussian kernel, and the cerebral cortex was parcellated based on gyral and sulcal information derived from manually traced brains (21, 26). These procedures are well-prescribed in a related paper (27).

### Statistical Analysis

Statistical analyses for demographic data were performed with R software (version 2.15.3). Assumptions of normality were tested for continuous variables using the Kolmogorov–Smirnov test; all demonstrated a normal distribution. Two sample *t*-tests and chi-square (χ^2^) tests were used to probe for differences between the male (*n* = 41) and female groups (*n* = 80) in terms of demographic variables, clinical data, and total intracranial volume (ICV). All statistical analyses used a two-tailed level of 0.05 for defining statistical significance.

FreeSurfer software (Version 7.1.0, http://surfer.nmr.mgh.harvard.edu/) was used for group analysis. Surface-based normalization was computed to map cortical volume data for each subject onto a common group space to allow comparison across subjects at homologous points on the cortex. Cortical anatomy measurements were then smoothed with FWHM = 10 mm, and the GLM was fit to the data with age, considering the *APOE* ε4 allele and total ICV as covariates. The vertex-wise statistical test was performed to evaluate the impact of the interaction between years of education and sex on cortical volumes. The uncorrected significance map with *p* < 0.001 was then overlapped onto the fsaverage brain template surface for visualization with a cluster-extent threshold of 100 mm^2^. In addition, we performed the False Discovery Rate (FDR) procedure for the multiple comparison correction (corrected *p* < 0.05). Furthermore, we performed multiple linear regression analyses to evaluate the effect of adjusted cortical volumes in regions of interest-by-sex interaction on neuropsychological test scores with a backward stepwise regression analysis (*p* < 0.05). Adjusted cortical volume = raw cortical volume – β × (intracranial volume [ICV]–mean ICV), where β is the regression slope of the region of interest ICV vs. neuropsychological test scores (28).

## Results

### Baseline Demographic and Clinical Data

Table 1 shows the baseline demographic and clinical data for the male and female groups. There were no significant differences in age, years of education, and *APOE* ε4 genotype between two groups. In regard to the neuropsychological battery, female group showed higher scores of CERAD-K WLM, WLR, and TM than the male group. In addition, the male group exhibited larger total intracranial volume than the female group.

**Table 1:** Demographic and clinical characteristics of cognitively normal older adults.

	**Male (*N* = 41)**	**Female (*N* = 80)**	***p-*value**
Age (years)	70.7 ± 7.6	68.2 ± 6.9	0.080
Education (years)	14.5 ± 3.3	13.4 ± 2.8	0.062
*APOE* ε4 carrier (%)	10 (24.4%)	21 (26.2%)	0.999
MMSE-K	27.9 ± 1.7	28.4 ± 1.2	0.090
CERAD-K WLM	18.6 ± 3.3	20.5 ± 3.3	0.003
CERAD-K WLR	6.3 ± 1.9	6.9 ± 1.5	0.048
CERAD-K WLRc	9.4 ± 0.9	9.5 ± 0.8	0.775
CERAD-K TM	34.3 ± 5.2	36.9 ± 4.9	0.008
CERAD-K Total	74.3 ± 8.8	76.4 ± 8.9	0.215
Total ICV (mm^3^)	1570369.8 ± 134957.5	1391004.9 ± 108680.7	<0.001

*Data are presented as mean ± standard deviation (SD) unless indicated otherwise. MMSE-K, The Korean version of Mini-Mental Status Examination; CERAD-K, Korean version of Consortium to Establish a Registry for Alzheimer's Disease; MMSE, Mini-Mental Status Examination; WLM, Word List Memory; WLR, Word List Recall; WLRc, Word List Recognition; TM, total scores of memory domains including CERAD-K, WLM, WLR, and WLRc scores; Total, total scores of CERAD-K; ICV, intracranial volume*.

### Sex-By-Years of Education Interaction for Gray Matter Volumes

**Figure 1:**
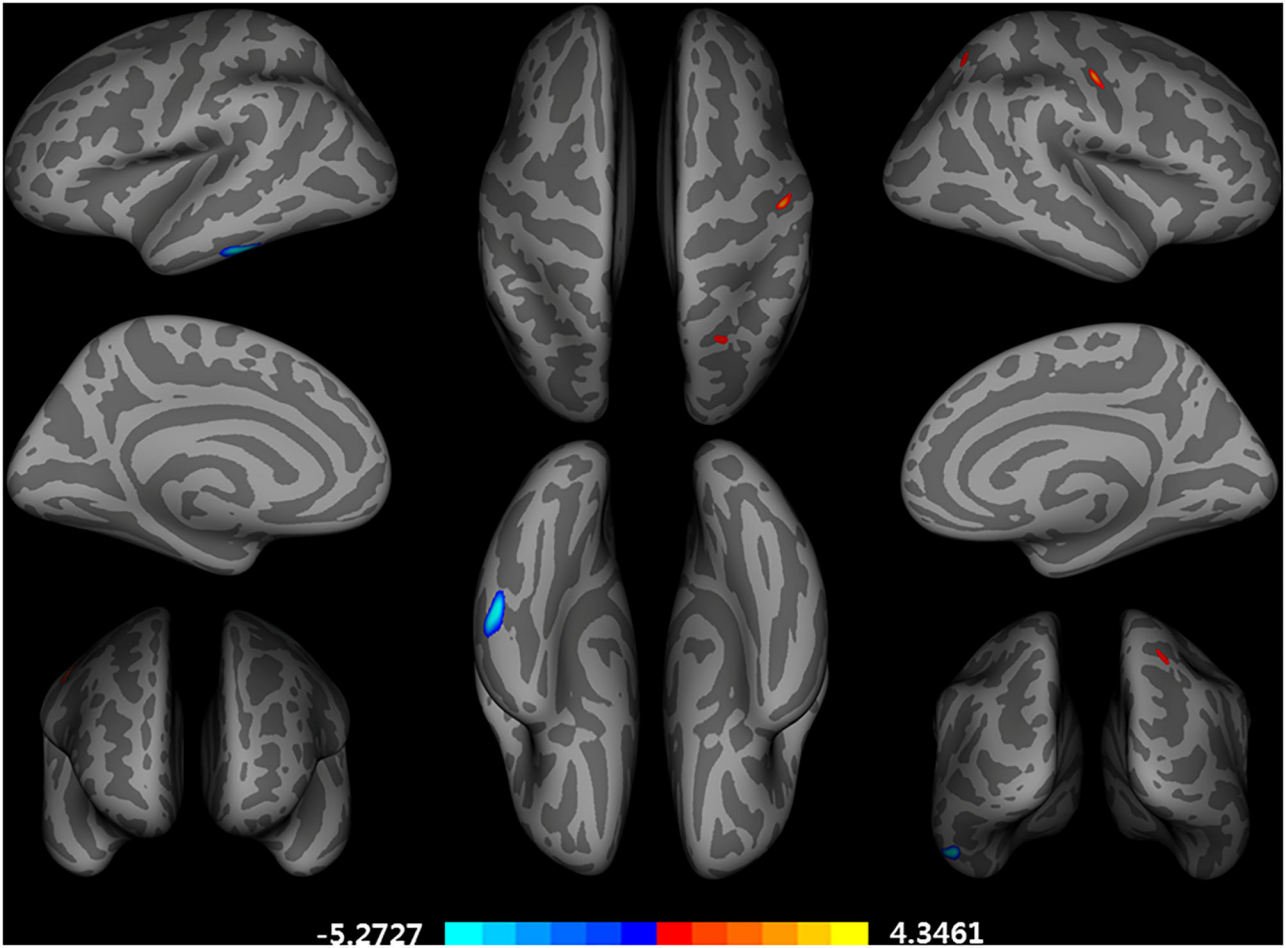
Brain regions with a significant impact of an interaction between years of education and sex on a cortical volume, adjusted for age, *APOE* ε4 allele, and total intracranial volume (uncorrected *p* < 0.001, false discovery rate-corrected *p* > 0.05). General linear model using QDEC tool available in the Freesurfer software. Blue color represents a negative association of an education year with cortical volumes in female participants and a positive association in male participants. Red color represents an opposing association with that described by red color.

**Figure 2:**
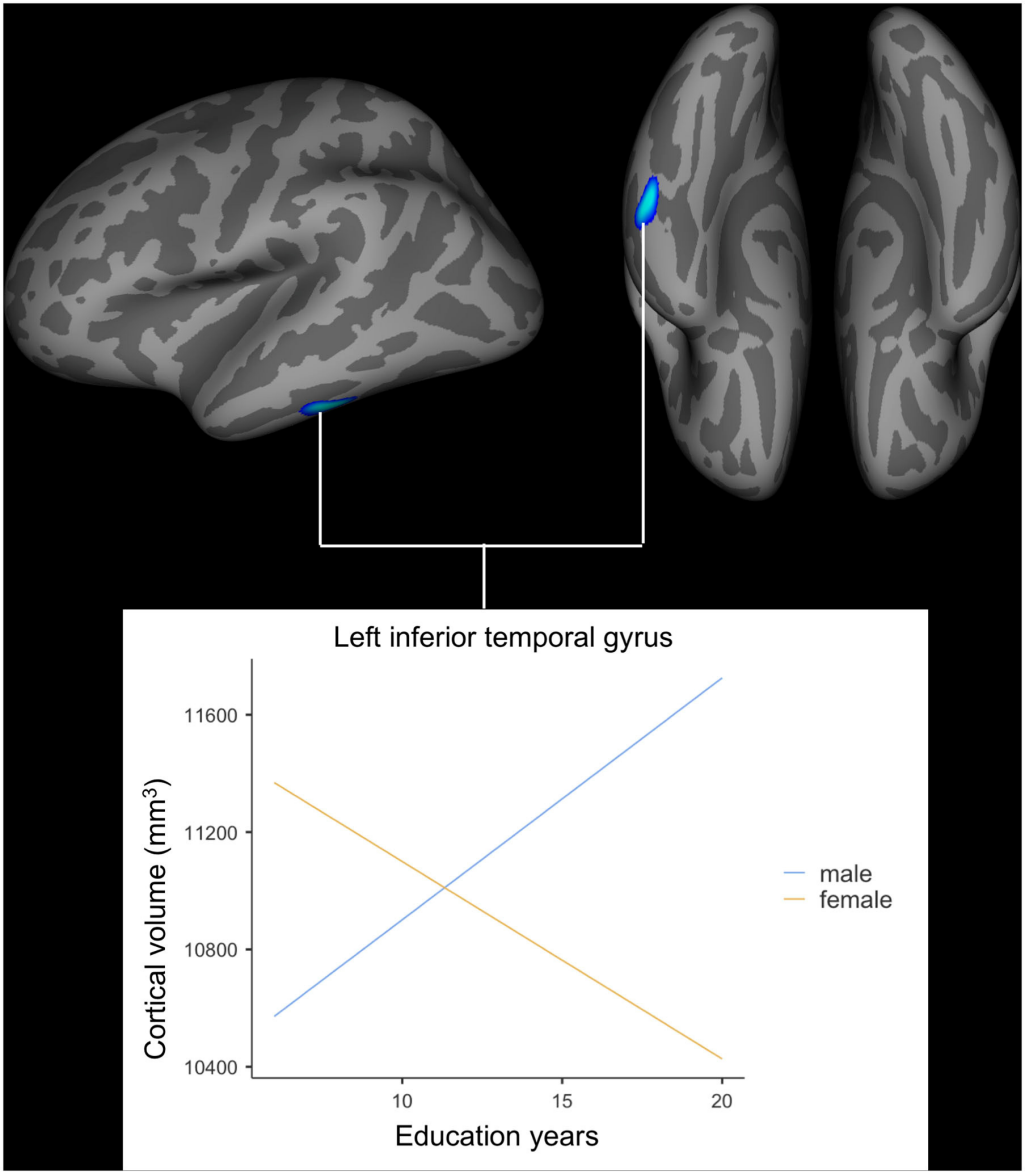
Impact of interaction between years of education and sex on cortical volume of the left inferior temporal gyrus. General linear model using QDEC tool available in the Freesurfer software. Uncorrected *p* < 0.001, false discovery rate-corrected *p* > 0.05.

After adjusting for age, *APOE* ε4 genotype, and total intracranial volume, the interaction between educational status and sex demonstrated a significant effect on the cortical volume of the left inferior temporal gyrus, right postcentral gyrus, and right superior parietal lobule (Figure 1). However, only the cluster size of the left inferior temporal gyrus exceeded 100 mm^2^ (Table 2). None of these interactions survived FDR correction for multiple comparisons. In addition, the regression slopes between years of education and cortical volume differed between the sexes. As expected from the visual inspection of the results shown in Figure 2, higher educational level was correlated with the greater cortical volume of the left inferior temporal gyrus in the male group. However, in the female group, the higher years of education were associated with the decreased volume of the left inferior temporal gyrus.

**Table 2:** Brain regions significantly impacted by the interaction between years of education year and sex on a cortical volume, adjusted for age, *APOE* ε4 allele prevalence, and total intracranial volume (uncorrected *p* < 0.001, false discovery rate-corrected *p* > 0.05).

**Brain region**	**Number of vertices**	**Size(mm^**2**^)**	**Max**	**Vertex Max**	**Tal X**	**Tal Y**	**Tal Z**
Left inferiortemporal gyrus	357	226.67	−5.2727	25467	−52.8	−36.7	−27.2
Right postcentralGyrus	203	74.24	4.3461	47225	40.5	−18.9	34.5
Right superiorparietal lobule	111	44.50	3.5525	74193	22.7	−59.5	49.9

*Number of vertices, number of vertices in cluster; Max, maximum voxel-wise significance in the cluster; Vertex Max, vertex number of the maximum; Tal, Talairach space coordinate*.

### Gray Matter Volume-By-Sex Interaction for Cognitive Functions

**Figure 3:**
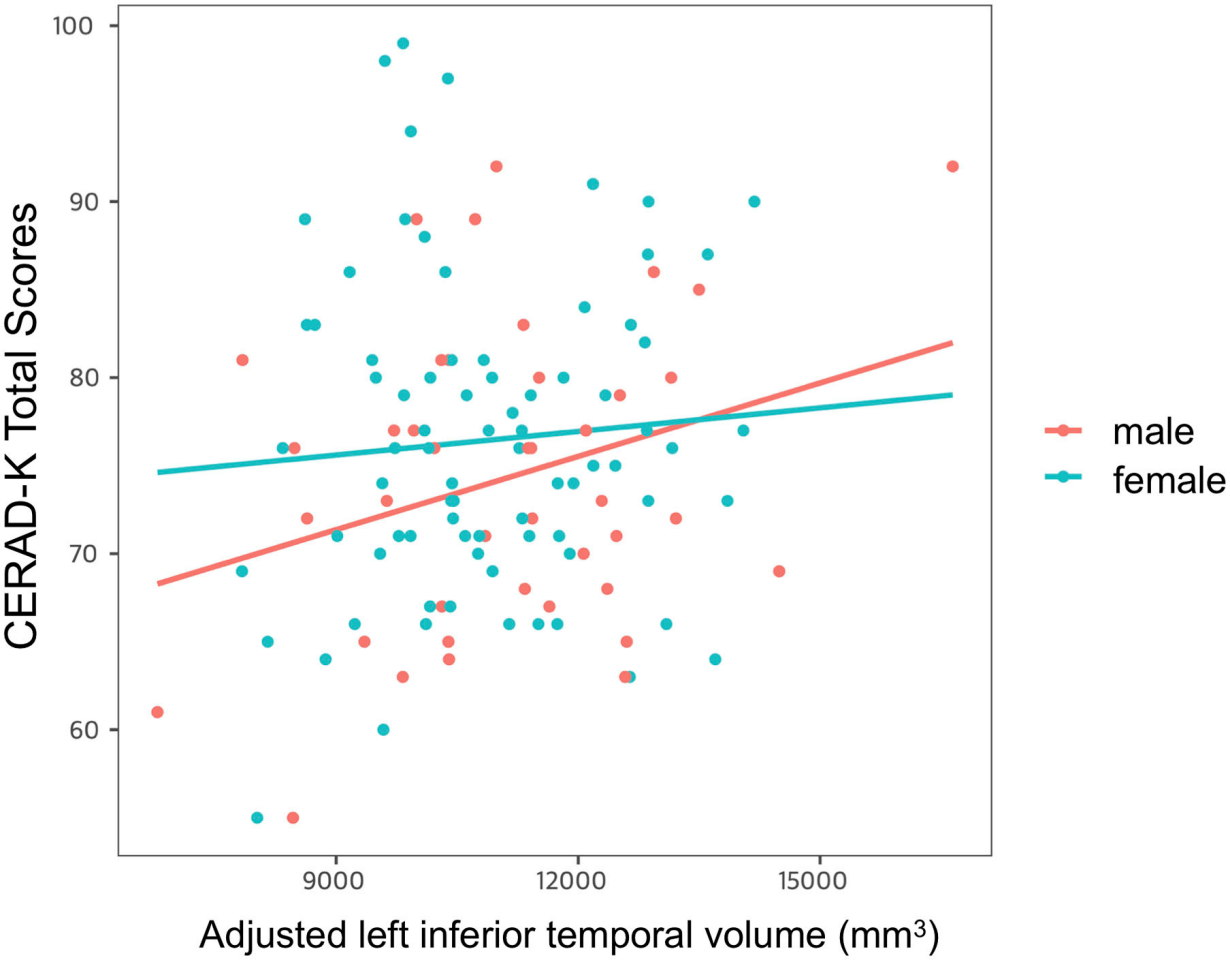
Impact of interaction between adjusted left inferior temporal volume and sex on cognitive function. Multiple linear regression analysis was used to predict the impact of interaction between adjusted left inferior temporal volume and sex on cognitive function after adjusting for age, years of education, and *APOE* ε4 allele prevalence. Stepwise backward elimination and all subsets regression were performed to select final model.

After controlling for age, years of education and *APOE* ε4 allele presence, we found a significant impact of interaction between adjusted left inferior temporal volume and sex on CERAD-K total scores (Figure 3). However, there was no main effect of interaction on other memory domains of the CERAD-K.

## Discussion

The present study aimed to evaluate whether the association between years of education and gray matter volume differs depending on sex in cognitively normal older adults. In addition, this study was designed to examine the brain regions that show years of education-by-sex interaction for gray matter volume through whole-brain analysis based on surface-morphometry.

The current paper found that the relationship between educational status and the cortical volume of the left inferior temporal gyrus differed significantly according to sex in cognitively healthy older adults. In older male participants, the correlation slope between years of education and cortical volume was positive, however, this slope was negative for older female participants. Although the cluster size was <100 mm^3^, the interaction found in the right postcentral gyrus and superior parietal lobe was observed in the opposite direction compared to that of the left inferior temporal gyrus. While the left inferior temporal gyrus has been reported to be generally tolerant to healthy aging (29) yet vulnerable to AD pathology (30, 31), the postcentral gyrus and superior parietal lobe have been demonstrated to show accelerated loss of cortical volume more than global atrophy present during the aging process (32). In this regard, the findings of this study suggest that greater years of education are associated with lesser cortical volume in brain regions that are relatively sensitive to AD pathology compared to the healthy aging process for older female participants.

Although the present research does not evaluate the presence of Aβ and tau retention, older female participants with healthy cognition have displayed a higher prevalence of Aβ and tau deposition than males between the ages of 60 and 85 (33). Additionally, prior studies have indicated that cognitively normal older adults with Aβ accumulation show greater atrophy in the hippocampus and supramarginal gyrus compared to those without Aβ retention (14). According to the cognitive reserve hypothesis, older adults with higher years of education could tolerate more AD-induced pathological change through compensatory mechanisms than those with comparable cognitive status (14). With the aforementioned propositions, it is estimated that more prevalent brain pathological changes in AD-vulnerable regions of older female subjects could have an effect on the negative association between years of education and cortical volume in the female group of the current study. Moreover, the inferior temporal gyrus is a Braak stage IV area, where neurofibrillary tangles associated with Aβ retention and cognitive impairment are accumulated (34, 35). Previous evidence has also demonstrated the relationship between this aforementioned tauopathy and cortical atrophy in the aging brain (36). Therefore, it is necessary to interpret the results of this study through the application of the cognitive reserve hypothesis for the two representative pathologies of AD. Since these pathologies were not evaluated in this study, additional studies to complement them should be conducted to enhance our understanding of the differential role of education in cortical atrophy according to sex.

Another important finding uncovered by the present study was the significant interaction between the inferior temporal gyrus volume and sex for global cognitive function in cognitively normal older adults. In a more detail, the cognitive function scores decreased more in the male group than in the female group when the cortical atrophy in the left inferior temporal gyrus progressed. This finding is consistent with the previous research, confirming that male sex is related to worse memory in cognitively intact older adults (37). Given that the inferior temporal gyrus is more vulnerable to AD pathology than to normal aging (30, 31), the compensatory process through cognitive reserve might be more activated to delay cognitive decline in females with relatively high AD pathology. However, as mentioned above, since AD pathology was not evaluated in this study, attention should be paid to interpretation. In addition, further research should be conducted to evaluate a brain functional change, which is closely related to AD pathology and cognition (38, 39).

Finally, several limitations need to be considered. Firstly, lifestyle factors (alcohol consumption, smoking, physical exercise etc.) (40, 41) and vascular risk factors (42), which might show differences between male and female subjects, could influence brain structure. In particular, physical exercise has been reported to increase the size of hippocampus, improve memory performances, and delay the onset of dementia (43, 44). In this regard, physical exercise has been also suggested as another proxy for cognitive reserve (3). Therefore, the possibility that these factors may have influenced the results of this study cannot be excluded. Secondly, this study evaluated only years of education as a proxy for cognitive reserve. Therefore, an additional study to comprehensively evaluate other brain/cognitive reserve proxies, including the aforementioned lifestyle and vascular risk factors, is necessary for further understanding.

The purpose of the current study was to determine the effect of education years-by sex interaction on gray matter volume by whole-brain analysis in cognitively intact older adults and to evaluate the distinctive sex-dependent impact of cortical volume on cognitive function. This study has identified the relevancy of the interaction between education and sex for gray matter volume of AD-vulnerable regions and has shown the differential impact of cortical volume on cognitive function according to sex. These findings have significant implications for the understanding of how sex may affect the role of cognitive reserve in differential cortical atrophy for cognitively intact older adults. In addition, alongside the supplementation of the aforementioned limitations, this research serves as a base for the deeper understanding of cognitive reserve in the aging and neurodegenerative disease processes.

## Data Availability Statement

The raw data supporting the conclusions of this article will be made available by the authors, without undue reservation.

## Ethics Statement

The studies involving human participants were reviewed and approved by the Institutional Review Board of The Catholic University of Korea. The patients/participants provided their written informed consent to participate in this study.

## Author Contributions

DK, S-MW, CL, and HL conceived and designed the research. DK, S-MW, H-RN, NK, and HL recruited subjects, and followed subjects to get clinical results. NK performed the *in vivo* MRI experiments. DK and S-MW performed the image preprocessing and image analysis. DK, S-MW, and NK performed statistical analysis. DK wrote the manuscript. CL and HL provided scientific mentorship throughout the project. All authors discussed the results and commented on the manuscript.

## Conflict of Interest

The authors declare that the research was conducted in the absence of any commercial or financial relationships that could be construed as a potential conflict of interest.
