# Supplementary material for: Differential Impact of Education on Gray Matter Volume According to Sex in Cognitively Normal Older Adults: Whole Brain Surface-Based Morphometry
Source: Front Psychiatry. 2021 Mar 5;12:644148. doi: 10.3389/fpsyt.2021.644148 (PMC7973038; doi:10.3389/fpsyt.2021.644148)
Supplement: Supplementary file 1 [file Data_Sheet_1.docx]

Supplementary Material

**Differential impact of education on gray matter volume according to sex in cognitively normal older adults: whole brain surface-based morphometry**

**Dong Woo Kang^1^, Sheng-Min Wang^2^, Hae-Ran Na^2^, Nak Young Kim^2^, Hyun Kook Lim^2^, Chang Uk Lee^1^***

^1^Department of Psychiatry, Seoul St. Mary’s Hospital, College of Medicine, The Catholic University of Korea, Seoul, Republic of Korea

^2^Department of Psychiatry, Yeouido St. Mary’s Hospital, College of Medicine, The Catholic University of Korea, Seoul, Republic of Korea

*** Correspondence:**Chang Uk Lee, MD, PhD

Department of Psychiatry, Seoul St. Mary’s Hospital, College of Medicine, The Catholic University of Korea, 222, Banpo-daero, Seocho-gu, Seoul, 06591, Republic of Korea

Tel: +82-2-2258-6082, Fax: +82-2-536-8744, E-mail: [jihan@catholic.ac.kr](mailto:jihan@catholic.ac.kr)

# Supplementary Methods

## Neuropsychological evaluation

# Cognitive status was assessed by neuropsychological testing at Seoul St. Mary’s Hospital, The Catholic University of Korea. The cognitive functions of all the subjects were assessed with the Korean version of the Consortium to Establish a Registry for Alzheimer’s Disease (CERAD-K), which included Verbal Fluency (VF), the 15-item Boston Naming Test (BNT), MMSE-K, Word List Memory (WLM), Word List Recall (WLR), Word List Recognition (WLRc), Constructional Praxis (CP), and Constructional Recall (CR). The CERAD is the standardized clinical and neuropsychological assessment battery for the evaluation of patients with Alzheimer's disease. The results were reviewed by a neuropsychologist to determine whether there was evidence of cognitive impairment.

# The VF score is the number of animal names that the subject could name in one minute. The BNT score ranges from 0 to 15 points. The MMSE-K score ranges from 0 to 30 points. The WLM score ranges from 0 to 30 points. The WLR score ranges from 0 to 10 points. The WLR score ranges from 0 to 10 points. The WLRc score ranges from 0 to 10 points. The CP score ranges from 0 to 11 points. The CR score ranges from 0 to 11 points.

## *APOE* genotyping

DNA was isolated from blood using the QIAmp Blood DNA Maxi Kit protocol (Qiagen, Valencia, CA). Genotypes for two APOE SNPs, rs429358 (E*4) and rs7412 (E*2) were determined using TaqMan SNP genotyping assays (Applied Biosystems, Foster City, California).
